# *Commiphora myrrh*: a phytochemical and pharmacological update

**DOI:** 10.1007/s00210-022-02325-0

**Published:** 2022-11-18

**Authors:** Gaber El-Saber Batiha, Lamiaa Wasef, John Oluwafemi Teibo, Hazem M. Shaheen, Ali Muhammad Zakariya, Opeyemi Abigail Akinfe, Titilade Kehinde Ayandeyi Teibo, Hayder M. Al-kuraishy, Ali I. Al-Garbee, Athanasios Alexiou, Marios Papadakis

**Affiliations:** 1 Department of Pharmacology and Therapeutics, Faculty of Veterinary Medicine, Damanhour University, Damanhour, 22511 AlBeheira Egypt; 2 Department of Biochemistry and Immunology, Ribeirão Preto Medical School, University of São Paulo, Ribeirão Preto, São Paulo Brazil; 3 Department of Biological Sciences, Sule Lamido University, Kafin Hausa, Nigeria; 4 Department of Biochemistry, University of Ibadan, Ibadan, Nigeria; 5 Department of Maternal-Infant and Public Health Nursing, College of Nursing, Ribeirão Preto, University of São Paulo, Ribeirão Preto, São Paulo Brazil; 6 Department of Clinical Pharmacology and Therapeutic Medicine, College of Medicine, Almustansiriyiah University, Bagh-Dad, Iraq; 7 Department of Science and Engineering, Novel Global Community Educational Foundation, Hebersham, NSW 2770 Australia; 8 AFNP Med, 1030 Vienna, Austria; 9 Department of Surgery II, University Hospital Witten-Herdecke, University of Witten-Herdecke, Heusnerstrasse 40, 42283 Wuppertal, Germany

**Keywords:** *Commiphora myrrh*, Medicinal properties, Volatile/Essential oil, Anti-septic, Anti-inflammatory, Anti-parasitic

## Abstract

Medicinal plants have a long track record of use in history, and one of them is *Commiphora myrrh* which is commonly found in the southern part of Arabia, the northeastern part of Africa, in Somalia, and Kenya. Relevant literatures were accessed via Google Scholar, PubMed, Scopus, and Web of Science to give updated information on the phytochemical constituents and pharmacological action of *Commiphora myrrh*. It has been used traditionally for treating wounds, mouth ulcers, aches, fractures, stomach disorders, microbial infections, and inflammatory diseases. It is used as an antiseptic, astringent, anthelmintic, carminative, emmenagogue, and as an expectorant. Phytochemical studies have shown that it contains terpenoids (monoterpenoids, sesquiterpenoids, and volatile/essential oil), diterpenoids, triterpenoids, and steroids. Its essential oil has applications in cosmetics, aromatherapy, and perfumery. Research has shown that it exerts various biological activities such as anti-inflammatory, antioxidant, anti-microbial, neuroprotective, anti-diabetic, anti-cancer, analgesic, anti-parasitic, and recently, it was found to work against respiratory infections like COVID-19. With the advancement in drug development, hopefully, its rich phytochemical components can be explored for drug development as an insecticide due to its great anti-parasitic activity. Also, its interactions with drugs can be fully elucidated.

This review highlights an updated information on the history, distribution, traditional uses, phytochemical components, pharmacology, and various biological activities of *Commiphora myrrh*.

**Graphical abstract:**

Graphical summary of the phytochemical and pharmacological update of *Commiphora myrrh*

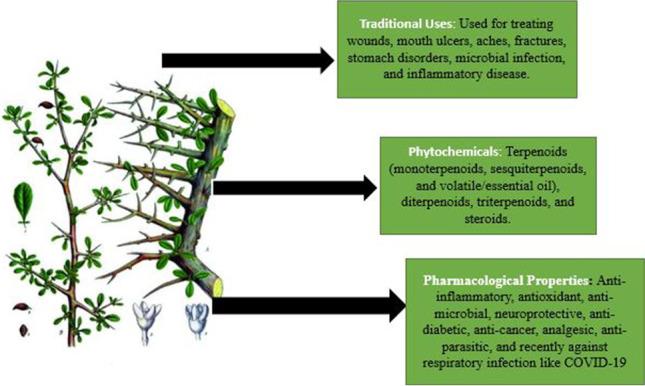

## Introduction

Resinous extract of plant origin has been regarded as an important plant resource in traditional medicine. Their effects and applications in traditional medicine are obtainable in Egyptian, Roman, Greek, and Chinese literatures. These extracts include frankincense, myrrh, benzoin, Dragon’s blood, and ferulae resin (Brand and Zhao 2017). Species of Commiphora exist as small trees or shrubs having rough and thorny branches. The species *Commiphora myrrh* found in the southern part of Arabia to the north-eastern part of Africa (mainly Somalia) and the north-eastern part of Kenya is responsible for the production of the true myrrh. Other types of Commiphora species that produce resins are found in Sudan, south of Arabia, Eritrea, Kenya, Ethiopia, and Somalia (Hanus et al. 2005b).

Myrrh, which has its origin from Arabia, is an extract that is produced by secretory tissues found on the bark of Commiphora species. Commiphora is from the family of Burseraceae which has more than 150 species of plant spread across subtropical and tropical regions, particularly north-east Africa, southern Arabia, and India (Demissew 1993). Species of the genus Commiphora are defined as small trees or shrubs having spinescent branches and bark that has pale-gray discharge or a reddish-brown resin. Resins produced by Commiphora have applications in fragrances, bouquet and as ointment in embalmment process, while their therapeutic applications have been gaining recognition in gradation among mankind. They are reported as having applications in traditional system of medicine for the management of all manner of ailments like wounds, ache, joint inflammation, fractures, parasitic infection, obesity, and gastrointestinal diseases (Abdul-Ghani et al. 2009a). Different types of bioactive constituents such as steroids, terpenoids, flavonoids, lignans, sugars, etc. were reported from members of the genus, Commiphora (Hanuš et al. 2005). Biological activities including anti-inflammatory, antimicrobial, antiproliferative, cardiovascular, and hepatoprotective activities associated with the crude extracts/pure compounds have been reported (Shen and Lou 2008). Reviews on the hypo-lipidemic activity of guggul, a resin from *Commiphora mukul*, was reported (Sahni et al. 2005). *Commiphora molmol* resin has been particularly applied in Egypt as an anti-parasitic drug, and the therapeutic applications were recently outlined (Abdul-Ghani et al. 2009a; Shen et al. 2012). The hypolipidemic property of guggul-sterol (Sharma et al. 2011), their molecular targets (Shishodia and Aggarwal 2004), as well as the secondary metabolites found in the genus of *Boswellia* and *Commiphora* were described (Shen and Lou 2008).

## Myrrh

Myrrh can be defined as an oleo-gum resin produced by different *Commiphora* species. It is constituted by 3–4% impurities, 7–17% volatile oils, 25–40% alcohol soluble resins, and 57–61% water soluble gum (Massoud et al. 2001). Myrrh resin constituents soluble in alcohol are commiphorinic acids, commiphoric acids, commiferin, heerabomyrrhols, and heeraboresene (Rao et al. 2001). Moreover, these resins have been reported to contain commiphorinic acid, α-, β-, and γ-commiphoric acids, commiferin, α- and β-herrab-omyyhols, cholestrerol, heerboresene, compesterol, kerto steroids, β-sitosterol, 3-epi-α-amyrin, and α-amyrone (Rao et al. 2001). One triterpenoid each was reportedly isolated from *Commiphora incisa* resin and *Commiphora kua* resin, the importance of each in chemotaxonomy was equally emphasized (Provan and Waterman 1986). The volatile oil fraction was reported to be composed of various bioactive constituents including elemol, eugenol, esters, cinnamaldehyde, cadinene cumicalcohol, cuminaldehyde, m-cresol, dipentene, limonine, pinene, sesquiterpenes, furano-sesquiterpenes heerabolene, and terpenes, (Rao et al. 2001), alcohols, α-camphorarene, myrcene, Z-guggulsterol, aldehydes I, II, III guggulsterol (Treas and Evans 1978). Galactose, alongside with acidic polysaccharide arabinose, 4–0-methyl-glucuronic acid, and xylose (2:7:8 with 18–20% protein) (Bisset and Wichtl 1994), is the major composition of the gums (soluble in water) or mucilage fraction. Hydrolyzing the gum gives galactose, 4–0-mythylglucuronic acid, arabinose, and xylose (Leung 1980). The major composition of terpenes in myrrh is furano-sesequiterpenoids with elemane, eudes-mane, guaiane, or runcates, structures (Bisset and Wichtl 1994). It has a peculiar odor resulting from furano-sesquiterpenes (Bruneton 1995), which is likely to be a significant constituent of pharmaceutical myrrh (Wichtl 1994). Solvent extraction using 90% alcohol from gum myrrh yielded 27–60% crude polysaccharide (PS) as reported by Evans and Trease (2002). Crude polysaccharide from myrrh was reportedly constituted of 18% protein (Anderson et al. 1965).

**Fig. 1:**
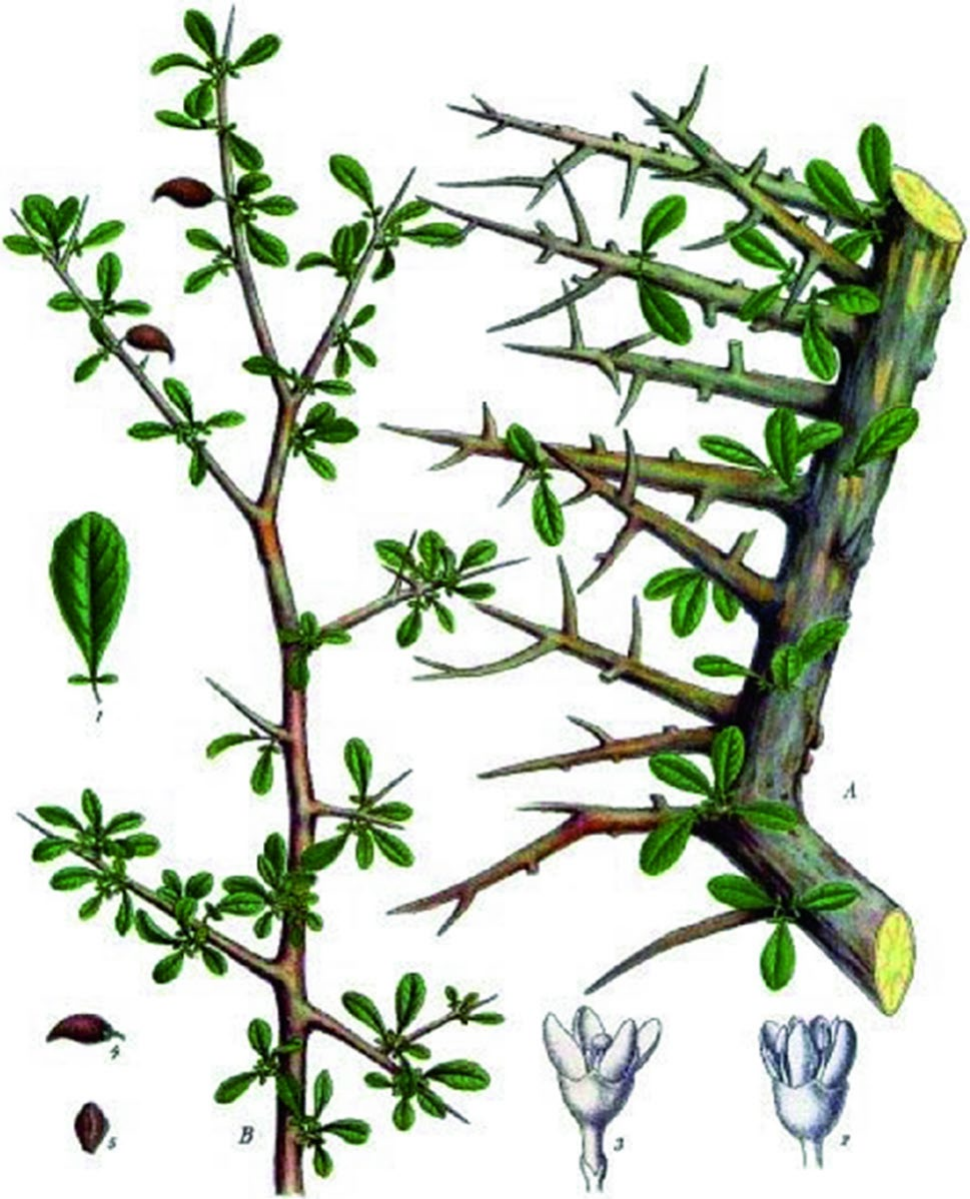
*Commiphora myrrha* (Akbar 2020)

Myrrh can be regarded as a transduce from *Commiphora*, family Burseraceae, tree bark (Fig. 1). This genus is made up of over 150 species, having its distribution in the arid, tropical and subtropical regions (Tucker 1986). The species, *Commiphora myrrha* (Nees) Engl., is responsible for the production of the true myrrh. This species has other synonyms; *Balsamodendron myrrha* Nees or *C. molmol* Engl *Commiphora myrrha* is used since time immemorial for treating wound and other medicinal uses (Shen et al. 2012). Its bioactive constituents such as furanodienes curzerene and lindestrene furanoeudesma-1,3-diene are sources of the fragrance emitted by myrrh and of its analgesic property (Dolara et al. 1996). Myrrh is recognized for its therapeutic properties since the ancient times and has been used extensively in the treatment of wounds, management of aches, inflammation of the joints, parasitic infections, obesity, and gastrointestinal diseases in the ancient Egypt (Abdul-Ghani et al. 2009b). It is made up of essential oils, gum (30–60%, water-soluble), myrrhol (3–8%, ether-soluble), and resin, myrrhine (25–40%, alcohol-soluble). The aromatic and transuding resins produced by the stem-bark of *Commiphora* species (Burseraceae family) are characterized as yellowish to reddish brown powder having a nicely bitter taste with a slight irritable balsamic smell (Hanus et al. 2005a).

## History of *Commiphora myrrh*

**Fig. 2:**
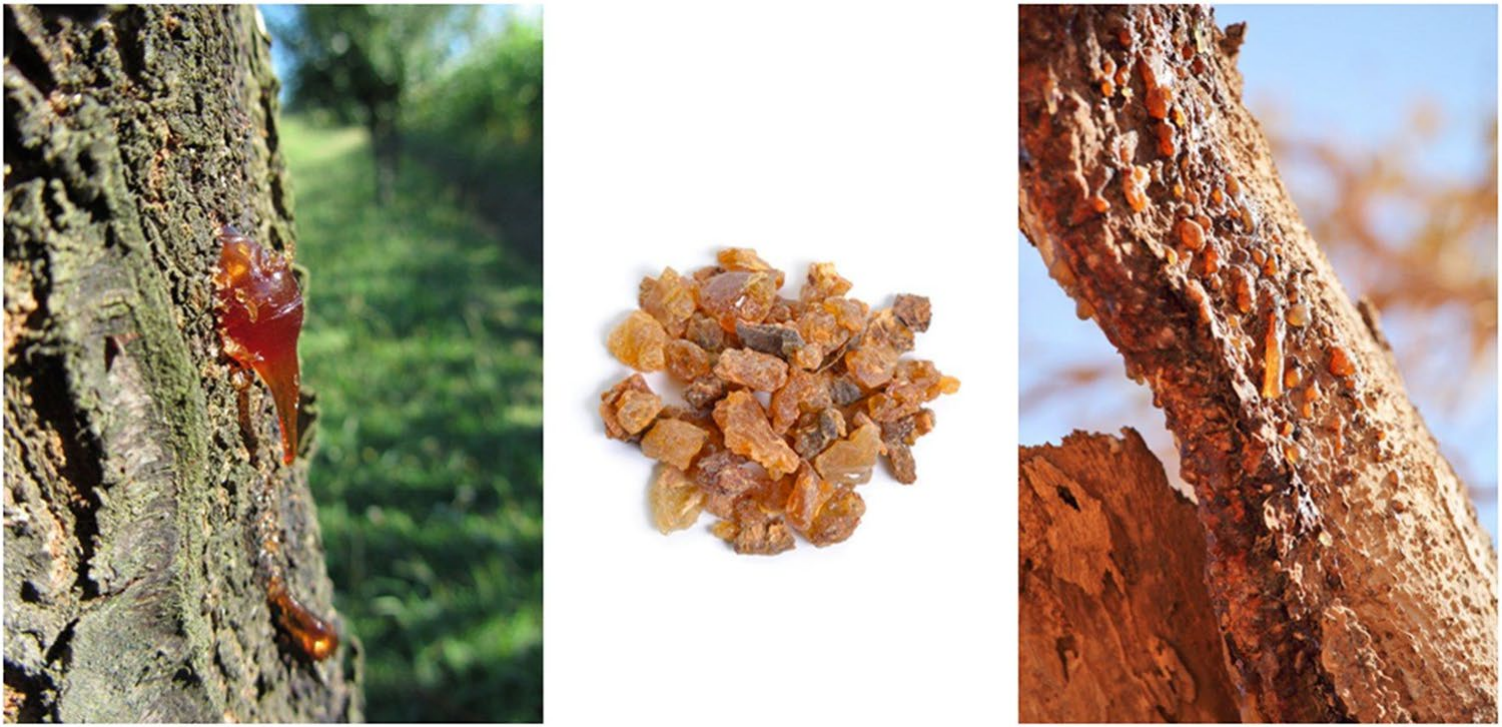
*Commiphora myrrha*, gum in nature (Alyafei, 2020)

*Commiphora genus is from Commiphora myrrh* tree. It is a flower producing plant classified into Burseraceae family (Alyafei, 2020). *Burseraceae* with origin from the arid, tropical, subtropical, and arid regions has species close to 150 (Gadir and Ahmed, 2014). The term “Myrrh” was coined from “murr” that has its origin from the Arabic language, meaning bitter. Traditionally, it is called *C. myrrh*, *Commiphora molmol*, *or Balsamodendron myrrha*, in the Greek and Chinese literatures (Germano et al., 2017). Myrrh, in combination with water, forms an emulsion, composed of 2–8% volatile oil, 23–40% resin (myrrhin), and 40–60% gum (Shameem, 2018) (Fig. 2). Myrrh essential oils are used as an adorning agent, incense, and bioactive agents. These oils are mainly made up of terpenoids and terpenes. In addition, myrrh possesses active ingredients that are of pharmaceutical importance, such as sesquiterpene lactones, used in the treatment of some ailments (Singh 2015).

## Traditional uses

The most studied and most frequently used species of *Commiphora* are *C. molmol, C. myrrha*, *C. mukul*, and *Commiphora opobalsamum*. These species resins have shown an array of pharmacological activities in treating wound, mouth ulcer, aches, fracture, stomach disorders, microbial infection, and inflammatory disease. In *Unani* medicine, gums are used as antiseptic, astringent, anthelmintic, carminative, emmenagogue, expectorant, and stomachic. They are also applied in combination with other drugs in preventive medicine against epidemic diseases and used ectopically in gouty and painful joints, and for the treatment of wounds. Sesquiterpenoids having an array of pharmacological properties were resin extracts menthofuran and furanoeudesma 1,3-dieneare known as major components in myrrh oil. In rats, myrrh hydro suspension can protect the gastric mucosa against various ulcer inducing agent, while the ethanolic extract was reported in mice to be anti-inflammatory against acute and chronic inflammation. (Akbar 2020). In a study on Egyptian patients infected with tinidazole and methronidazole resistant *T. vaginalis*, administration of Myrrh between 6 and 8 consecutive days showed significant improvement in the patients’ health condition. In another study, 85% of the patients infected with fascioliasis were cured after treatment with 600 mg dose for a period of six consecutive days. Several studies have also documented over 90% recovery from schistosomiasis in patients from Egypt. In Italy, RCT of myrrh extract in patients experiencing aches from various etiologies, like fever- associated ache, joint ache, lower back ache, muscle aches, headaches, and dysmenorrhea, was observed to have significantly alleviated the aches (Akbar 2020). The medicinal importance of the myrrh, guggulin, a resin from an Indian *C. mukul*, in Ayurvedic medicine dates as far back as 3000 years ago. This resin has its application in the enhancement of bone fracture healing, mouth ulcer, sore throat, wound healing, acne, skin disorders, lymphadenopathy, and intestinal worms, as documented in Bhava Prakasha’s Materia Medica (Shilpa and Venkatesha Murthy, 2011). A variety of sesquiterpenes with different pharmacological activities have been reported from the resin (Xu et al. 2011). Menthofuran and Furanoeudesma 1,3-diene were shown in report as the main bioactive compounds present in myrrh oil. Ethanol extract was reported to majorly consist of curzerene, 2-tert-butyl-1,4-naphthoquinone, benzenemethanol, and 3-methoxy-α-phenyl (Mahboubi and Kashani 2016).

Earliest antimicrobial use of myrrh by Sumerians for the treatment of teeth infection and intestinal worms’ dates as far back as 1100 BC. Another ancient use of myrrh was in embalming by the Egyptians in ancient times. Also, its oil has for long being used in treating *Candida albicans*, *Tinea pedis* fungal infections, and subcutaneous wound (Stevensen 1998). *The documentations from British Herbal Pharmacopoeia* (de Rapper et al. 2012) showed myrrh tincture usage as a mouthwash in gingivitis and ulcers. Organizations such as the European Commission ((Blumenthal 1999) approved the use of myrrh in the management of mild inflammation (topically) of the pharyngeal and oral mucosa. In Chinese medical system, myrrh is also regarded as an important drug for curing syphilis, leprosy, and rheumatism (Nomicos 2007). A decoction of myrrh is traditionally used in the treatment of stomach ache in Somalia and Ethiopia (de Rapper et al. 2012).

Terpenoids have been shown to exhibit different pharmacological activities together with anti-molluscic (Borkosky et al. 2009), hypoglycemic (Agwaya and Nandutu, 2016), anesthetic (local) (Tsuchiya and Mizogami 2017), cytotoxic (Shoaib et al. 2017), and microbicidal effects (Zengin and Baysal 2014).

Other diseases such as aches, arthritis, and inflammatory diseases have been treated (Ding and Staudinger 2005). In Egypt, *C. molmol* resin is being marketed as an anti-parasitic agent under a brand name Mirazid (Abdul-Ghani et al. 2009a). In Arabian medical system, myrrh is reportedly applied in the stomach and inflammatory disease management (Al-Harbi et al. 1997). Myrrh has long been employed in China as far back as the Tang Dynasty (600 AD) as documented in the Chinese medical literature, Hai YaoBen Cao; resins from *C. Myrrha* or *C. opobalsamum* is the commonest resin used in China. Usually, it is co-administered with frankincense management of trauma, aches, swelling, inflammation of the joints, and fractures (Al-Bishri and Al-Attas 2013). It has a wide application in dermatology for the treatment of sores, skin ulcer, and empyrosis (Shen et al. 2012). The ability of myrrh to break up coagulated blood and promote circulation of blood is the bases for its usage in the treatment of arthritis, fracture, trauma as well as tumors (Singhuber et al. 2009; Yang 2009). China has now become the biggest myrrh importing country in the world, and its most application is in medicine (Coppen 1995).

In the bible, it was related that Christ was given myrrh at birth. Thus, it was rated higher costing much more than gold. The greatest of all medieval clinicians, al-Razi, a Muslim physician, used myrrh to treat diseases of the kidneys and bladder, and to dissipate swellings in the stomach.

Myrrh belongs to Burseraceae family, being a fragranced oleo gum resin produced as a transude from *Commiphora myrrha stem bark.* Reports have shown that it is an effective anti-parasitic and microbicidal agent with marked activities on glandular fever, gingivitis, mouth ulcers, sinusitis, brucellosis (Abdel-Hay et al. 2002). Furthermore, myrrh volatile oil and its crude extracts have shown various pharmacological activities including microbicidal, anti-inflammatory, cytotoxic, sedative actions (Massoud et al. 2004c).

Generally, the Chinese medical system suggested that myrrh has potent synergistic therapeutic activities like analgesic, anti-inflammatory, blood activation, and antibacterial effects upon use with frankincense (Sadowska-Bartosz et al. 2014).

## Pharmacology

In rats, an aqueous suspension of myrrh was reported to protect the gastric mucosa from various ulcerogenic agents (Al-Harbi et al. 1997). While in mice, ethanolic extract showed anti-inflammatory activity on acute and chronic inflammation (Atta and Alkofahi 1998). Mirazid, an oleo-resin formulation obtained from a purified oleo-resin extract, was shown to reduce parasite counts of *Giardia lamblia* by 100% in the intestine and feces of infected rats (Fathy 2011). In dermatology, essential oils of myrrh and ethanolic extracts inhibited the growth of these dermatophytes: *Microsporum gypseum*, *Microsporum canis*, *Trichophyton rubrum*, *Trichophyton mentagrophytes*, and *Trichophyton verrucosum* (including the oil was more potent) (Mahboubi and Kashani 2016). Aqueous resin inhibited the growth of *Enterococcus faecalis* in the tooth cavity; this activity was equated with 2% chlorhexidine, a standard drug. Myrrh protects basically by preventing hepatocyte alteration and significantly reducing the portal areas granulomas. It also ameliorates fibrosis intercellularly as observed in mice infected with bilharzia (Massoud et al. 2004b). A related form of protection has been observed with an Egyptian strain of *Schistosoma mansoni* infected mice (Massoud et al. 2005). At sub-toxic doses, myrrh oil was reported to have stimulated the production of IL-6 and IL-8 not through the epithelia cells but by human gingival fibroblasts. However, it reduced significantly the production of IL-1β (Tipton et al. 2003). In terms of toxicity, myrrh has been reported to be highly toxic on EAC cell-bearing mice, while its biological action on tumor cells was equated to CP (Ai-Harbi et al. 1994). Myrrh emulsion has shown good antioxidant potential and guards against hepatic oxidative damage and immunotoxicity on exposure to lead acetate by downregulating LPO and stimulating antioxidant and immune defense mechanisms (Ashry et al. 2010). Resins as supplement have been shown to greatly attenuate liver injury induced by ammonia and decreased ammonia circulation and TNF-*α* of hyper-ammonemic rats (Mahmoud et al. 2017a). About hepatocarcinogenesis in rats induced by DEN, resin extracts showed a significant decrease in tumor proliferation, circulating markers of inflammation, liver LPO, NO, and angiogenesis (Mahmoud et al. 2017b). However, biochemical parameters showed no improvement or delay in hepatocarcinogenesis induced by DEN (El-Shahat et al. 2012). A strong antithrombotic activity has also been reported by myrrh extract (Olajide 1999).

## Phytochemical studies

Studies have shown over 300 molecules from the genus *Commiphora*. Information on isolated compounds are as provided in Hanuš et al. (2005). This review is important as it presents findings of over a decade including phytochemical regularities of this genus. A broad summary of metabolite structures and resources under structural types and classification was provided by the Supplementary Data. On the aspect of phytochemicals from this genus, emphasis should be made on: (i) Terpenoids particularly those of the sesquiterpenoids and triterpenoids as they are the predominant bioactive constitutes of the genus. (ii) With regard to phytochemicals, more emphasis had been laid on *C. myrrha*, *C. kua*, *C. mukul*, *and C. confuse C. molmol species*. Commiphora species resins were administered based on recommendations of the traditional medicine systems of China, India, Egypt, etc. Hence, these genus resins are the most investigated product with a potential of discovering bioactive compounds.

Studies on the phytochemicals of resin from *C. myrrh* showed that it contains heerabolene, elemol, acadinene, cuminaldehyde, eugenol, multitude of furano-sesquiterpenes, including furanodienone, furanodiene, curzerenone, and lindestrene (Vishakha and Ramyasree 2015). Approximately 20 different types of furano-sesquiterpenoid compounds have been isolated and characterized from exudates of myrrh (Su et al. 2009b). Furthermore, two pure compounds, 2-methoxyfuranodiene and 2-acetoxyfuranodiene belonging to furano-sesquiterpenoid family, were isolated from *C. myrrh* gum (Maradufu 1982).

### Terpenoids

#### Monoterpenoids, sesquiterpenoids, and volatile oil

Monoterpenoids are seen majorly in volatile oils, characterized using the GC technique. Several studies have reportedly used GC analysis for the characterization of Commiphora species volatile oils, such as *C. myrrha* (Dekebo et al. 2002; Morteza-Semnani and Saeedi 2003), *Commiphora quadricincta* (Assad et al. 1997), *Commiphora holtziana* (Dekebo et al. 2002; Provan et al. 1987), *Commiphora guidottii* (Craveiro et al. 1983), *Commiphora kataf*, and *Commiphora sphaerocarpa* (Dekebo et al. 2002). Mono-terpenoids reported include camphene, myrcene, limonene α-pinene, and β-pinene. Observations on the constitution of volatile oils obtained from species of Commiphora varied significantly. In volatile oil, sesquiterpenoids having a lower degree of oxidation play an important role. Β-selinene β-Elemene, α-humulene, α-copaene, and germacrene B have been reported to be predominately distributed in the volatile oils from species of Commiphora. Abundance of furanosesquiterpenoids in the genus Commiphora can be used to define the genus. Elemanolide (4), guaianolide (5), and cadinanolide (6 and 7) isolated from C. myrrha and *C. opobalsamum* resin are the main bioactive constituents from the genus recently (Shen and Lou 2008; Shen et al. 2008, 2009).

#### Diterpenoids

*C. mukul* resin, which commonly has diterpenoids camphorene, was the first diterpenoid compound isolated from essential oil of *C. mukul* (Sarup et al. 2015). Similarly, these two compounds, cembrane and verticillane, are diterpenoids found in same species. A pimarane diterpenoids, aracopimaric acid (8), and two abietane diterpenoids, abietic acid (9) including dehydroabietic acid (10), were all *C. myrrha* isolate (Su et al. 2009a).

#### Triterpenoids

Triterpenoids were the most biologically active compounds identified in resin of species of Commiphora; however, flavonoids and lignans majorly occur in the plant stem (Shen et al. 2012). The main bioactive components in essential oils reported from different species of Commiphora were sesquiterpenes hydrocarbons, oxygenated sesquiterpenes, and monoterpenes, that is different in different species (Marongiu et al. 2005).

Triterpenoids constitute the largest number of bioactive constituents isolated and purified from species of Commiphora. They include cycloartane, dammarane, oleanane, octanordammarane, runca, anostane polypodane, and ursane. The largest group of triterpenoids are the dammarane with over twenty-one of them being identified in resins from four species including *C. confuse* (Manguro et al., 2003), *Commiphora dalzieli* (Waterman and Ampofo 1985), *C. myrrha* (Shen et al. 2009), and *C. kua* (Manguro et al. 2003), and *C. mukul* is the only species from the genus that produces the polypodane triterpenoids (Matsuda et al. 2004a, 2004b). Cycloartane triterpenoids (11–20) having new replacement at carbon (C-2) were reported from *C. myrrha* and *C. opobalsamum* (Shen et al. 2008).

#### Steroids

*C. mukul* are the only species that have produced nine cholestane steroids and eleven pregnane steroids. Also, isolated from *C. mukul* resin are two isomers of carbon (C 21) steroid, Z-, and E-guggulsterones (21 and 22) mukul (Patil et al. 1972). These compounds generated attention due to the potent antitumor, hypolipidemic, and anti-inflammatory activities they possess (Shishodia et al. 2008).

#### Miscellaneous

Among the Commiphora species are additional types of bioactive constituents including flavonoids, lignans, carbohydrates, and long chain aliphatic derivatives. The gum of Commiphora has been reported to consist of carbohydrate that basically exists as polysaccharide (Kumar and Shankar 1982). The gum yields mono or disaccharide on hydrolysis. Resins oozing out of Commiphora species are devoid of flavonoids unlike the flower, stem, and bark. Long chain aliphatic derivatives of 1,2,3,4-tetrahydroxy like guggultetrol-20 and D-xylo-guggultetrol-18 have been reported in the gum (Patil et al. 1972). They usually occur in nature as glycoside or runcat acid ester (Shen et al. 2007).

## Biological activities

### Anti-inflammatory activity

In Ayurvedic medicine, resin from *C. mukul*, sometimes refered as “guggul,” for centuries was used in arthritis treatment. Resin extract from *C. mukul* in a clinical study showed significant improvement in osteoarthritis after treatment with 500 mg TID for 1 month (Singh et al. 2007). A significant inhibition of NO formation by methanol resin extract of *C. mukul* in lipopolysaccharide (LPS) activated murine macrophages was exhibited using IC50 = 15 mg/mL (Meselhy 2003; Matsuda et al. 2004a). Also, MeOH extract demonstrated an anti-inflammatory property against LPS-induced inflammation (Cheng et al. 2011). Isolated compounds, polypodane triterpenoids, cembrane diterpenoids, lignans, and steroids have been studied for COX inhibitory activity and NO production. Prevention of NO production by E and Z-guggulsterones (22 and 21), myrrhanol A (23), and myrrhanone A (24) has been observed having IC 50 = 1.1, 3.3, 21.1, and 42.3 mM, respectively (Meselhy 2003). In lipopolysaccharide activated mouse peritoneal macrophages, myrrhanol A (23) and mukulol (25) were reported to have inhibitory action nitric oxide synthase (iNOS) induction (Matsuda et al. 2004a). E-guggulsterone (22) and Cembrene (26) were reported to be the most active in COX inhibition. They inhibited COX-1 by 79% and 67%, and COX-2 by 83% and 54% individually at 100 ppm (Francis et al. 2004). The anti-inflammatory properties of Z-guggulsterones and E-guggulsterones (21 and 22) were reported, eliciting their anti-inflammatory activity by subduing NF-kB activation and NF-kB regulated gene products expression (Shishodia and Aggarwal 2004; Lv et al. 2008). Kimura et al. (2001) reported *C. mukul* resin extract and pure compounds anti-inflammatory potential (23 and 24) using the model, air pouch granuloma induced by an adjuvant and observed that 23 was 7 × , 5 × , and 3 × more effective on carmine content, granuloma, and pouch fluid weight more than the standard drug (hydrocortisone) used as control. It, therefore, has the prospect of being developed into an anti-inflammatory drug. Furthermore, myrrh was reported to have significant inhibitory activity on the activator of transcription-1, transcription-3 (STAT-1 and STAT-3), and signal transducer leading to reduced production of cytokines through the pathway of janus kinase/STAT (Lv et al., 2008). Also, it suppresses down-regulation of cytokine synthesis, a reaction to a decreased interferon-gamma and interleukin-beta production. This auto-regulates JAK/STAT pathway through the control of transcription by transcriptions activator that also inhibits activation of pathway (Su et al. 2011).

Su et al. (2012) reported that myrrh water extract and combined water extract (CWE) at doses of 3.9 g/kg and 5.2 g/kg exhibited formalin-induced paw edema inhibition with an inhibition rate of 30.44% and 23.50%. Individually, a significant (*P* < 0.01 or *P* < 0.05) inhibition of PGE_2_ production was observed in samples tested. However, CWE was stronger in suppressing carrageenan-induced mice paw edema at 2 and 3 h after drug was administered. Reports showed the anti-inflammatory activities of extracts from *C. molmol* and *Commiphora pyracanthoides*. Inhibiting the release of IL-6 and IL-8 stimulated by IL-b in human gingival fibroblasts cells stimulated IL-b on the administration of *C. molmol* volatile oil has also been reported (Tipton et al. 2003). *C. molmol* resin pet. ether extract inhibited carrageenan-induced inflammation and cotton pellet granuloma. Extract from the stem of *C. pyracanthoides* was reported to be the most active with an IC50 = 27.86 mg/mL among all the Commiphora species tested for anti-inflammatory activity applying a 5-lipoxygenase (5-LOX) assay method (Paraskeva et al. 2008). Friedelin, an isolated from *Commiphora* berryi and its pet. ether bark extract, was reported to have shown inhibitory activity on soybean lipoxygenase with IC50 values of 35.8 mM and 15.3 mg/mL (Kumari et al. 2011). *Commiphora erythraea* resin hexane extract was reported to inhibit edematous response, reducing it by 84% at 1000 mg/cm^2^ in mice ear edema caused by croton oil. These compounds, Myrrhone (27), rel-3R-meth-oxy-4S-furanogermacra-1E,10 (15)-dien-6-one (28), and rel-2R-methoxy-4R-furanogermacr-1(10)E-en-6-one (29) present in the extract were proposed to have exhibited the anti-edematous activity of the plant (Fraternale et al. 2011). Ethanol leaf extract of *Commiphora caudate* administered orally at 250 mg/kg was reported to have inhibited carrageenan-induced paw edema response by 67% in rats (Annu et al. 2010). In vivo anti-inflammatory activity of isolated compounds, mansumbinoic acid (30) and 2a,3b,23-trihydroxyolean-12-ene (31), were reportedly studied (Fourie and Snyckers 1989; Duwiejua et al. 1993). Evidence from literatures showed *C. mukul* resin was the most investigated, showing promising anti-inflammatory property both in vitro and in vivo. This further establishes application in Ayurvedic medicine. Steroids (21 and 22) and triterpenoids (23 and 24) present in *C. mukul* are the active principles bringing about anti-inflammatory effects. The extracts and compounds mechanistic activity from the genus Commiphora as it relates to signaling pathways, multiple inflammation-related proteins were highlighted. Potential anti-inflammatory targets such as NO formation, COX, ROS, TNF-a, PGE2, MAPK, and NF-kB were identified and tested.

### Antioxidants

Compounds of the class diterpenes, sesquiterpenoids, sterols, and triterpenes present in high quantity in myrrha extracts that may serve as electron donors react with free radicals converting them to a more stable product thereby terminating the radical chain reactions. This is a corroborated (Fraternale et al. 2011) research work where they showed myrrha resin hexane extract as having the best DPPH radical scavenging activity unlike to its oils. The same authors made suggestion that the action could be attributed to three compounds, 2-methoxy-furanogermacren-6-one myrrhone and 3-methoxy-furano germacradien-6-oneall of the furano-sesquiterpenoids family. Their DPPH radical scavenging potential had IC 50 values of 1.08, 4.29, and 2.56 mg/mL, respectively (Mohamed et al. 2014).

Triterpenes (ursolic and oleanolic acid) and essential oils in the resins of *C. myrrh* and *Boswellia serrata* were reported as having potent antioxidant activity in sunflower oil, although, with negative result in DPPH scavenging activity. *C. myrrha* essential oil (EO) inhibited lipid peroxidation in sunflower oil. It is then safe to conclude that essential oil of *C. myrrh* could be applied in functional foods, pharmaceutical, and cosmetic preparations mainly due to their antioxidant activity in oil substrate (Assimopoulou et al. 2005).

### Antimicrobial activity 

Biological activity of myrrh on viruses and bacteria has been reported in literatures. Empirical evidences have shown that myrrh extracts possess effects on virus by virtue of which these extracts possess antibacterial and antiviral activities on different virus strains (Khalil et al. 2020). In a particular study, bactericidal, fungicidal, and anti-viral activities of myrrh essential oil extracts suggested their potential in inhibiting the growth of bacteria and virus strains (Brochot et al. 2017). Also, in a study, essential oils from myrrh showed antiviral activities against two viruses: herpes simplex virus type 1 (HSV-1) and influenza virus type A (H1N1). Myrrh was observed to act by free viral particles direct inactivation and disrupting the virion envelope structures which major role is in host cell virus invasion (Brochot et al. 2017). Another mechanism by which the extracts bring about their activity is by the inhibition of the enzyme, DNA polymerase in viral strains, thereby, hampering virus resistance to specific medications. Therefore, development of new antiviral drugs from the extracts with specific target on DNA holds a lot of prospects (Brochot et al. 2017).

Generally, plants Eos are a mixture of different constituents (Burt 2004). Specific compounds from phenols were suggested to show the microbicidal activities of Eos (Ben Arfa et al. 2006; Lambert et al. 2001). Also, four terpene molecule activities present in some Eos were investigated on the food pathogens and spoilage bacteria organisms. Since disruption of cellular membranes was reported to be caused by Eos and their constituents (Kapros and McDaniel 2009), studies on their cytotoxicity have been carried out using bacteria cell model in vitro (Boffa et al. 2016).

Studies on PE myrrh extract using diffusion test showed antimicrobial potential on *C. albicans*, *Streptococcus pyogenes*, and *Staphylococcus aureus*. The extract of EtOH showed potent action against the strains tested. However, greater activity was observed against *C. albicans* and *S. aureus* (9 mm zone of inhibition, 20 mg /mL); this further establishes the therapeutic effect of myrrh for curing infectious diseases such as gingivitis pharyngitis, phyorrhoea, and sinusitis (de Rapper et al. 2012). The anti-fungal activity exhibited on *C. albicans* is similar with the findings reported in previous studies (Dolara et al. 2000). Methanol extract tested on *C. albicans, Pseudomonas aeruginosa*, and *Escherichia coli* demonstrated a very low antimicrobial activity with no zone of inhibition observed at 20 mg/mL, whereas, PE extract demonstrated 3.7 and 5.7 mm zone of inhibitions for *C. albicans* and *S. albus*, which can be compared to 5 and 3 mm for *S. aureus* (Boffa et al. 2016).

### Neuroprotective effects

From the resins oozing out of *Commiphora myrrha* was isolated runcate type sesquiterpenes, i.e., commiterpenes A–C (1–3) showing neuro-protective activity on MPP^+^ induced neuronal cell death in SH-SY5Y cells (Xu et al. 2011).

Commiphoins A–C (1–3), the novel runcate type of sesquiterpenes, including two common runcate type of sesquiterpenes (4 and 5) were gotten the extracts of *Commiphora myrrha* resinous. Screening 1 and 3–5 compounds was carried out against anti-Alzheimer’s disease (AD) activity employing *Caenorhabditis elegans* AD pathological model. All the compounds tested demonstrated significant anti-Alzheimer’s disease activities (Yu et al. 2020).

### Anti-acetylcholinesterase activity

Acetylcholinesterase inhibitors, natural or synthetic, have been shown to be commonly applied as insecticides or nootropic drug for boosting memory in patients having amnesia. Many bioactive constituents have been established to have the ability to inhibit AchE which helps in boosting cerebral activity or ameliorate disease symptoms relating to it (Teibo et al. 2020). Herbal preparations with known activities on the brain for boosting retention and learning are referred to as “nootropic herbs” or “phyto-nootropics,” and their isolated active principles are called smart drugs (Hussein et al. 2019). In Mesopotamia, the species commonly used to produce essential oils used in aromatherapy is *Commiphora myrrha* (Nees), Engler (Watt and Sellar 2012). *C. myrrha* leaves, bark, and resin methyl alcohol extract have been reported to inhibit AchE by 17.00, 26.00, and 29.33% compared to eserine. Computational prediction using silico tools has been used to model the ADMET and putative anticholinesterase potentials of bioactive compounds in myrrh. Bioactive constituents from *C. myrrha* were reported to show a good binding affinity (BA) concerning AchE principal sites with a range of − 5.8 (m-cresol) to − 10.5 (abietic acid) kcalmol^−1^. On these bases, terpenoid compounds (25 out of 28) from myrrh served as dual inhibitors due to the hydrophobic interactions using both peripheral anionic site (PAS) of AchE, and catalytic triad while hydrogen bonding was used between AchE and alpha-terpineol, elemol, and eugenol (Hussein et al. 2019).

### Stimulates insulin secretion

Medicines of plant origin like *Commiphora myrrha* (CM) have traditional application in Ayurvedic medicine for diabetes management in certain regions of Africa and Arabia. Several studies have shown that in diabetic animal models, CM reduced blood glucose, and increased insulin concentration is achieved with CM. It is, however, not fully clear the mechanism employed by CM in achieving glycemic control in the animals (Al-Romaiyan et al. 2021).

Increase in insulin production that was concentration dependent was observed on exposure of MIN6 cells to CM resin solution (0.5–10 mgmL^−1^) in a static setting. When islet of the mouse was incubated with CM (0.1–10 mgmL^−1^), it brought about a concentration-dependent stimulatory effect on insulin. Reduction in cell viability or cell membrane integrity was not associated with CM concentrations at ≤ 2 mgmL^−1^. Although, a remarkable absorption of trypan blue dye and apoptosis accompanied higher concentrations of CM. At stimulatory and sub-stimulatory glucose levels, CM (2 mg/mL) resulted in quick and reversible increase in insulin production by islets of both humans and mouse during perfusion Total insulin contained in β-cells, mRNA formulations of preproinsulin, and Pdx1 did not change despite the stimulating effect of CM on the production of insulin (Al-Romaiyan et al. 2021).

### Analgesic action

In ancient times, myrrh has been used as analgesics, which is possibly due to bioactive constituents present in them acting as pain relievers (El Ashry et al. 2003). Two sesquiterpenoid compounds, furanocudesma-1, 3-diene, and curzerene present have been reported to be acting on the receptors opioid in the central nervous system, bringing about anesthetic activity (El Ashry et al. 2003). Also, furanocudesma-1, 3-diene in myrrh, particularly the ones isolated from *Commiphora mukul* have been reported to provide significant relief from abdominal pain and improving health hyperalgesia. Hence, these extracts bring about their effects by relieving peripheral nerve pain resulting from chronic compressive damages to the sciatic nerves. These extracts have been reported to be applied as a substitute medication in the management of nerve pain (Mehta and Tripathi 2015). In addition, some isolate such as furanocudesma-1, 3-diene and lindestrene present in myrrh were reported to relief pain by acting on nerves and body joints. These compounds bring about their effects by suppression of the molecule prostaglandin and hinder the inward movement of sodium current thereby ameliorating the feeling of pain (Nomicos 2007). The presence of the compound furanodiene in high amount acts by lowering pain resulting from fever (Gadir and Ahmed 2014).

### Anti-cancer property

Cell deaths occur in various form, one of which is apoptosis (cell suicide), defined as a programmed process or genetically controlled, or necrosis or a non-programmed/accidental process (Hotchkiss et al. 2009). One of the most effective non-surgical cancer treatments is targeting apoptosis, a characteristic of cancer cells. Targeted attack on apoptosis holds the possibility of stopping the uncontrolled growth of the cancer cells. Studies have reported the use of cancer drugs to target different pathways of apoptosis (Pfeffer and Singh 2018), including compounds of plant origin and having various bioactive compounds, is having an effect on the apoptotic pathways via different mechanisms (Safarzadeh et al. 2014; Teibo et al. 2021a, b). The use of flow cytometry to detect necrosis or apoptosis by exposing phosphatidylserine (PS) outside of the apoptotic cells, an important method in the induction of apoptosis, has been reported (Wlodkowic et al. 2011). Another important discovery in the study of cancer is the inability to control the normal cell cycle. There is an increasing interest on the cell cycle as a mechanism for anticancer drug target (Gabrielli et al. 2012). In a study where flow cytometry was used for detecting the activity of certain compounds on HepG2 cell cycles, it was shown that treated HepG2 cells in the S-phase reduced in percentage, while an increase was observed in the G2/M phase cells. In a research work, cell cycle phase distribution using flow cytometry was used to determine the altered compound in HepG2 cells cell cycle. The results showed decrease in treated HepG2 at the S phase; however, that of the G2/M phase increased.

Ethno-pharmacological evaluation has identified myrrh an anticancer drug. The active constituent possessing anticancer activity is elemene with proven action on various cancerous cells including glioblastoma, and it was proven to be safe and effective. Elemene, particularly β-elemene, was reported to possess anti-proliferative activity. It acts by activating p38MAPK in glioblastoma (Yao et al. 2008). Also, the compound 2S-epoxy-4R-furanogermacr-10-3n-6-one, furose-type sesquiterpene rel-1S isolated from myrrh was reported to possess low cytotoxic activity on MCF-7 cell line of breast cancer. This compound, in combination with bisabolene in myrrh, was effective in reducing the growth of breast cancer indicating myrrh as containing novel anti-breast cancer drug (Yeo et al. 2016; Shen et al. 2008). Cyclobolinane, a triterpenoid present in myrrh, was shown to exert a moderate cytotoxic effect on PC3 and DU145 prostate cancer cell lines (Shameem 2018).

Furthermore, numerous studies reported the use of myrrh components in apoptotic induction and stoppage of tumor cell proliferation (Chen et al. 2013). This is similar to reports on compounds isolated from *C. myrrh* as used to induce apoptosis and arrest of cell cycle progression (Gao et al. 2015).

Furano-sesquiterpene compounds were shown to elicit some pharmacological activities resulting from the wide range of bioactive compounds present in them (Fraternale et al. 2011). The derivatives of furano-sesquiterpenes and itself, a soft coral isolate, were reportedly tested on leukemia, prostate, lung, breast, and cervix cancer cell lines which resulted in some of the compounds possessing promising activity on two of the cancer lines tested, leukemia and prostate cancer (Rajaram et al. 2013). Additionally, a different furano-sesquiterpene reportedly obtained from soft coral was tested for its anticancer activity. Researchers have demonstrated the inhibition of many cancer cell line proliferation in humans, decreased programmed cell death, and cell cycle arrest induction in human leukemia cells (THP-1) (Arepalli et al. 2009).

**Fig. 3:**
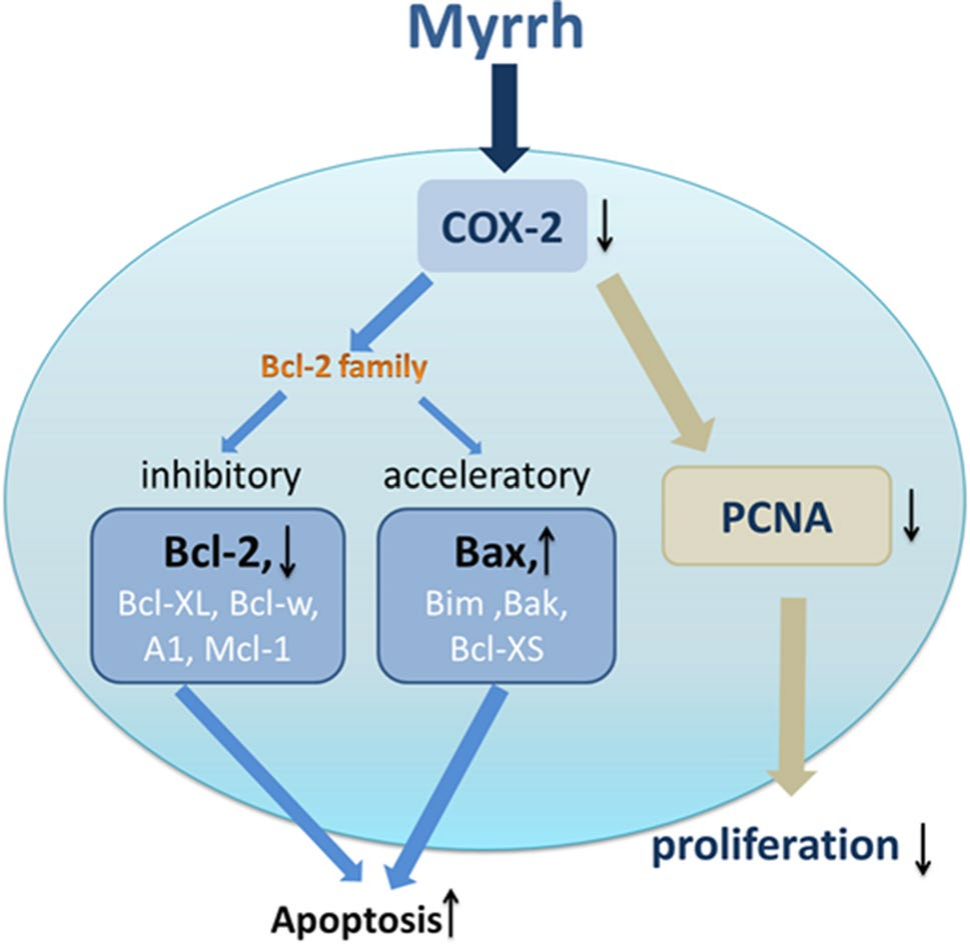
Myrrh’s inhibition of proliferation of gastric cancer cells and induction of apoptosis (Sun et al. 2020)

Results from different researches have suggested that myrrh possesses inhibitory properties against cell multiplication and brings about cell suicide of GC cells possibly achieved by downward control of COX-2 formulation in GC cells (Sun et al. 2020) (Fig. 3). Below is a schematic representation of relevant proteins showing concurrent upward control of Bax formulation, downward control of COX-2, and Bcl-2 formulation in cells during myrrh administration. In vitro studies have shown myrrh to induce apoptosis in a dose-dependent form in GC cells. GC cell migration rate could be possibly reduced by myrrh. Although, blockage of GC cell migration could increase with increase in administration time.

### Antiparasitic activity

In Egypt, the use of myrrh as an anti-parasitic agent saw its revolution in 1990 through evidence-based scientific research. The main focus of research in Egypt was the human trematode infection shrouded in stories of success and disagreement.

#### Myrrh as a schistosomicide

Myrrh has been reported to have schistosomicidal activity on different phases of *S. mansoni*. The effect of the drug in infected mice was more pronounced at the 21^st^ and 45^th^ days of PI. The drug showed a promising prophylactic effect when administered 5 days before exposure (Massoud et al. 2004c). A unique myrrh formulation that contains volatile oil and myrrh resin was recorded at the beginning of the 2000s for its efficacy and safety in mice with *S. mansoni* infection. Extract of myrrh, administered at 250 mg/kg and 500 mg/kg, was described to have stimulated a noticeable reduction in worm burden, increasing hepatic displacement of worms and a radical reduction in immature egg percentages situated on the intestine wall (Badria et al. 2001). Mirazid was reported in mice model to be safe in the treatment of Schistosoma infection by *S. mansoni*, and it was also efficacious (Hamed and Hetta 2005). An improvement in liver enzyme activities and a significant worm load reduction by 81.10% and a reduction in ova count by 73.07% on daily administration of Mirazid, for 3-day fasting at 600 mg/kg (Hamed and Hetta 2005). In another study, myrrh was reported to have shown significant schistosomicidal activity at 500 mg/kg on administration every day for five consecutive days. The activity remained pronounced in groups that got the drug on the 21^st^ and 45^th^ days post infection (Massoud et al. 2004c). Furthermore, it was observed in the groups a significant reduction in granulomas restored jejunal mucosa continuity and led to paucity of eosinophils (Massoud et al. 2004c). However, results from other studies have reported otherwise the chances of myrrh application for the management of schistosomiasis. The most interesting report on inefficacy of myrrh on animals infected with *S. mansoni* was from a multi center research carried out by Botros et al. (2004). Mirazid, a commercial formulation, has been tested alongside myrrh resin derivatives at varying doses on various strains. In the Egyptian (CD) strain infected mice reduction in worm infection loads in mice was observed to be negligible. Solution of Mirazid, at high doses, was observed to be toxic to mice infected with Puerto Rican (Mill Hill) strain of *S. mansoni* but possessed modest to no worm reduction at lower doses. With Puerto Rican (NMRI) and Brazilian (LE) strains of *S. mansoni* mice and hamsters, no anti-schistosomal activity was observed. Furthermore, effects on tissue oogram and egg arrangement did not show significance (Botros et al. 2004)*.*

#### Myrrh as a fasciolicide

Contrary to the inconsistent activity on schistosomiasis infection, myrrh has proven to possess a very potent fasciolicidal activity. This is evident from the significant data generated on this activity from experimental animals, infield studies, and clinical trials.

Myrrh showed high efficacy when tested on Fasciola in animal studies. It was reported in a study, that Mirazid completely eradicated Fasciola gigantica in rabbits with 20 mg/day dosage administered orally for six days consecutively. The immune response (IgG) was observed to be highest in infection treated rabbits compared to the infection untreated control (Mahmoud 2008). This lead to the conclusion, that at parasitology and immunology levels, Mirazid was safe and the most effective fasciolicidal drug (Mahmoud 2008).

#### Myrrh as a heterophycide

Heterophytes are common parasite found on snails and fish serving as intermediary hosts, found along the Nile valley and lakes of Egypt reportedly subjected to myrrh. In animal and clinical studies, both showed promising results in terms of efficacy on the parasite. In an animal study, emulsion form of Mirazid showed significant activity on heterophyidiasis with very high reduction in load of worm, tegumental spines disfigurement, and attrition as seen under a scanning electron microscope. It was observed to be very active, leading to 100% worm load reduction at 500 mg/kg/day administered for consecutively for 3 days (Abdul-Ghani et al. 2009a).

#### Myrrh as a molluscicide

Aside from its reported termiticidal activity, myrrh extract has been reportedly tested on snail intermediate hosts of trematodes for activity. Based on the reports of it being safe and having activity on the parasite and its intermediate hosts, this provides it with the benefit of being effective in the control and treatment of diseases. It has been investigated the anti-molluscic potential on Egyptian snail species *Bulinus truncates*, *Lymnaea cailliaudi*, and *Biomphalaria alexandrina*. These snails’ species and their eggs were subjected to the drug at different conc. over a period of 24 and 48 h at 22–26 °C. The outcome of the experiment was *B. alexandrina* had an LD50 and LD90 (155 ppm and 195 ppm), higher than B. runcates (50 ppm and 95 ppm) and *L. cailliaudi* (50 ppm and 85 ppm) on 24-h exposure. A mortality rate of 100% for egg clutches of *B. alexandrina* and *L. cailliaudiat* was recorded at 100 ppm and 75 ppm respectively. However, in order to obtain similar results on 48 h of exposure, lower concentrations were required. Under laboratory conditions, reports on myrrh showed significant inhibitory effect on snail intermediate hosts, especially their eggs. On snail hosts of schistosomes, myrrh extract showed mulluscicidal activity. After 24 h of post exposure, molluscicidal activity on *B. alexandrina* and *Biomphalaria alexandrina*, at concentrations 20 and 10 ppm respectively, was reported (Massoud and Habib 2003). Prolonged exposure was observed by authors to have resulted to increase in infected snail’s number. Ovicidal activity of myrrh has been reported to be effective on a day-old egg mass of snail than on 5-day-old snail egg mass. Also, the eggs demonstrated more resistance to the drugs than adult snails.

#### Against respiratory infections 

Myrrh from *Commiphora* have been reported as having activity on sore throat and chest infection. It acts by subduing inflammatory responses associated with it (Khalil et al. 2020).

Extract of *C. myrrh* and its essential oil have been reported to be used as expectorants, essential for the management of respiratory diseases like chest infection and any ailment associated to it (Germano et al. 2017). Similarly, in myrrh the activity of aromatic gum resin has been reported infection in the chest (Su et al. 2015). The resin employs the anti-inflammation and cytotoxic mechanism on bacteria or fungi infection responsible for the chest ailment. Also, the *C. myrrh* extract and resin were reported to show analgesic and anti-inflammatory activity which further justifies their use as an essential herbal medicine for different chest pain (Su et al. 2011).

#### Nasal congestion effects

In infections relating to cold and flu, myrrh or *Commiphora myrrh* extract reduces nasal congestion. Myrrh resin serves as an immune-stimulant in the season of cold and flu by accentuating the immunological system and acting as an expectorant in nasal congestion treatment (Kalra et al. 2011). Traditionally, myrrh oil has been added to hot water in a few drops and inhaled in the form of steam. Headaches associated with nasal congestion have been treated with myrrh indicating its analgesic effect (Ferrara 2019)**.**

#### Can myrrh combat COVID-19?

It is a common belief that myrrh or *C. myrrh* is a medicinal plant having applications in the management of various diseases due to its therapeutic effects. Reported therapeutic properties of *C. myrrh* include analgesic, immunomodulation, cytotoxic, anti-inflammatory, antioxidant, hepatoprotective, antimicrobial, anti-tumor, and anti-ulcer properties. Hence, myrrh could be applied in the treatment and management of various diseases due to its diverse biological activities. Also, it has been reported to possess antiviral activities, hence, can be used in the prevention of various diseases. On this bases, it is could be possible to use myrrh or *C. myrrh* in the treatment of the most recent pandemic, COVID-19 (Alyafei 2020).

In states like Qatar, the sale of herbal preparations and myrrh has skyrocketed on emergence of COVID-19, so has charge increased. This trend could be associated to the traditional beliefs in the use of herbal medicines or that existing knowledge on the medicinal and/preventive properties of myrrh necessitated this, hence, the need for further investigation. It has been recently proposed that research into the activity of myrrh mouthwashes on COVID-19 could serve as likely source of a therapeutic agent against the disease (Alyafei 2020).

#### Toxicity

Essential oils of myrrh at high doses have been reported to produce adverse side effects in mice recently (Lamichhane et al. 2019). It has also been documented that women that applied substantial quantity of myrrh while pregnant had miscarriages (Al-Jaroudi et al. 2016). In patients with hot temperament, it is usually not recommended. There is dearth of information on the animal toxicity studies (Akbar 2020).

In terms of human consumption, myrrh, like most herbal and botanical products, is perceived as being harmless. Myrrh as certified by the US Food and Drug Administration is a natural flavoring agent (Ford et al. 1992). In a research safety of myrrh was determined and carried out by administering the agent orally at 11.5 mg/kg for three consecutive days after overnight fasting (Massoud et al. 1998). After 1, 2, 4, and 8 weeks posttreatment in individuals that were not infected, liver and kidney function tests were observed to be normal while no significant side effect was observed generally (Massoud et al. 1998). In a parallel study, 10 mg/kg was administered for 3 to 6 days endured with mild and transient detrimental side effects (Massoud et al. 2001). In another study, myrrh extract of 500 mg/kg was administered for five consecutive days but was observed to show no signs of hepatotoxicity in mice after 12 weeks of post treatment (Massoud et al. 2004a). Comparing genotoxicity, hepatotoxicity and carcinogenic outcomes of myrrh extract, Mirazid formulation and praziquantel (standard drug), in animal model (rat) using various markers such as bilirubin, ALT, and AST in the serum were assessed, as well as the liver histopathology and bone marrow cell cytogenetic studies. Myrrh administration was at 500 kg/kg daily, for 6 weeks, while the conventional drug, praziquantel, was administered at 1500 mg/kg weekly for six consecutive weeks (Omar et al. 2005). Praziquantel had hepatotoxic, genotoxic, and carcinogenic effects. However, Mirazid was observed to be safe, showing no signs of hepatotoxicity, genotoxicity, or carcinogenic effect (Omar et al. 2005). This report corroborates prior findings on the safety of myrrh even after prolonged usage. Mirazid solution showed acute toxicity in mice having an LD 50 of 3139 mg/kg. In mice, Mirazid solution showed 100% and 75% mortality against Puerto Rican *S. mansoni* strain after 36 h of exposure at a dose of 1000 mg/kg and 300 mg/kg respectively, for 3 days consecutively (Botros et al. 2004).

#### Potential drug-herb interaction

Co-administration of myrrh with cyclosporine was observed to have decreased the bioavailability significantly in rats (Al-Jenoobi et al. 2015).

## Conclusion

Resinous extract of plant origin has been regarded as an important plant resource in traditional medicine. Many of the biological activities of *Commiphora myrrh* include anti-inflammatory, antioxidant, anti-microbial, neuroprotective, anti-diabetic, anti-cancer, analgesic, anti-parasitic, and recently against respiratory infection like COVID-19. These pharmacological properties are owned due to various phytochemicals such as terpenoids (monoterpenoids, sesquiterpenoids, and volatile/essential oil), diterpenoids, triterpenoids, and steroids. Its essential oil has applications in cosmetics, aromatherapy, and perfumery. With the advancement in drug development, hopefully its rich phytochemical components can be explored for drug development especially as a formulation as insecticide due to its great anti-parasitic activity as well as its interactions with drugs can be fully elucidated.

Table of summary

**Table:** 

*Commiphora myrrh*	
Location	Southern part of Arabia, the northeastern part of Africa in Somalia and Kenya
Traditional uses	Used for treating wounds, mouth ulcers, aches, fractures, stomach disorders, microbial infection, and inflammatory disease
Phytochemicals	Contains terpenoids (monoterpenoids, sesquiterpenoids, and volatile/essential oil), diterpenoids, triterpenoids, and steroids
Application	Cosmetics, aromatherapy, and perfumery
Pharmacological properties	Anti-inflammatory, antioxidant, anti-microbial, neuroprotective, anti-diabetic, anti-cancer, analgesic, anti-parasitic, and recently against respiratory infection like COVID-19

## Data Availability

Data sharing not applicable to this article as no datasets were generated or analyzed during the current study.
